# **β**-Catenin mutations and aberrant nuclear expression during endometrial tumorigenesis

**DOI:** 10.1054/bjoc.2000.1581

**Published:** 2001-01

**Authors:** M Saegusa, M Hashimura, T Yoshida, I Okayasu

**Affiliations:** Department of Pathology, Kitasato University School of Medicine, 1-15-1 Kitasato, Sagamihara, Kanagawa, 228-8555, Japan

**Keywords:** β-catenin, mutation, endometrial carcinoma, atypical hyperplasia

## Abstract

To clarify the possible role of aberrant β-catenin expression during endometrial tumorigenesis, a total of 199 cases of endometrial carcinomas (endometrioid type), as well as 37 cases of simple/complex and 32 of atypical hyperplasias, was consecutively investigated for immunohistochemistry, along with 141 normal endometrial samples distant from carcinomas. Of 199 carcinoma cases, 73 tumours as well as 44 normal samples were also analysed using a combination of RT-PCR and Southern blot hybridization, Western blot, and mutation gene assays. Cell membrane β-catenin immunoreactivity showed a stepwise decrease from normal, through atypical hyperplasia, to grade 3 carcinomas. In contrast, the nuclear accumulation in atypical hyperplasias and grade 1 or 2 tumours was higher than in simple/complex hyperplasias. Mutations in exon 3 of the β-catenin gene involving codons 33, 34, 37, 41, and 45 were observed in 16 (22.9%) of 70 endometrial carcinomas, as well as 3 (12.5%) of 24 atypical hyperplasias, the results being significantly related to low membrane and high nuclear immunoreactivity but not relative mRNA expression levels, suggesting that the gene mutations may be closely associated with changes in subcellular distribution. In addition to significant association between β-catenin mutation and low grade histological malignancy (*P* = 0.048), the mutations were detected in none of 15 and 13 (26%) of 50 tumours with or without lymph node metastasis, the difference being significant (*P* = 0.027). These findings suggest that β-catenin abnormalities may play an important role in a relatively early event during the endometrial hyperplasia-carcinoma sequence. © 2001 Cancer Research Campaign http://www.bjcancer.com


*British Journal of Cancer* (2001) **84**(2), 209–217
© 2001 Cancer Research Campaign
doi: 10.1054/ bjoc.2000.1581, available online at http://www.idealibrary.com on IDEAL®

http://www.bjcancer.com

### β-Catenin mutations and aberrant nuclear expression during endometrial tumorigenesis

**M Saegusa, M Hashimura, T Yoshida and I Okayasu**

Department of Pathology, Kitasato University School of Medicine, 1-15-1 Kitasato, Sagamihara, Kanagawa 228-8555, Japan

**Summary** To clarify the possible role of aberrant β-catenin expression during endometrial tumorigenesis, a total of 199 cases of endometrial carcinomas (endometrioid type), as well as 37 cases of simple/complex and 32 of atypical hyperplasias, was consecutively investigated for immunohistochemistry, along with 141 normal endometrial samples distant from carcinomas. Of 199 carcinoma cases, 73 tumours as well as 44 normal samples were also analysed using a combination of RT-PCR and Southern blot hybridization, Western blot, and mutation gene assays. Cell membrane β-catenin immunoreactivity showed a stepwise decrease from normal, through atypical hyperplasia, to grade 3 carcinomas. In contrast, the nuclear accumulation in atypical hyperplasias and grade 1 or 2 tumours was higher than in simple/complex hyperplasias. Mutations in exon 3 of the β-catenin gene involving codons 33, 34, 37, 41, and 45 were observed in 16 (22.9%) of 70 endometrial carcinomas, as well as 3 (12.5%) of 24 atypical hyperplasias, the results being significantly related to low membrane and high nuclear immunoreactivity but not relative mRNA expression levels, suggesting that the gene mutations may be closely associated with changes in subcellular distribution. In addition to significant association between β-catenin mutation and low grade histological malignancy ($P = 0.048$), the mutations were detected in none of 15 and 13 (26%) of 50 tumours with or without lymph node metastasis, the difference being significant ($P = 0.027$). These findings suggest that β-catenin abnormalities may play an important role in a relatively early event during the endometrial hyperplasia-carcinoma sequence. © 2001 Cancer Research Campaign http://www.bjcancer.com

**Keywords**: β-catenin; mutation; endometrial carcinoma; atypical hyperplasia

β-catenin, a major component of the cadherin adhesion system, binds to both E-cadherin cytoplasmic and α-catenin amino-terminal domains (Ozawa et al, 1989; Gumbiner, 1996). Recent studies have revealed that β-catenin also acts as a down-stream component of the Wingless/Wnt signalling pathway in Drosophila (Peifer et al, 1992). Although the role of β-catenin in signal transudation is not fully understood, the free cytoplasmic type has been considered to be able to interact with DNA-binding proteins of the T cell factor-lymphoid enhancer factor (Tcf-Lef) family (Beherens et al, 1996; Molenaar et al, 1996).

The adenomatous polyposis coli (APC) protein regulates free β-catenin levels, together with glycogen synthetase kinase-3β (GSK-3β) via phosphorylation of multiple serine/threonine residues encoded by exon 3 of the β-catenin gene, through the ubiquitin-proteasome pathway. Increase of free β-catenin may result from either APC inactivation or β-catenin mutations, with oncoprotein action through β-catenin-Tcf-regulated transcription (Aberle et al, 1997; Morin et al, 1997; Orford et al, 1997). A possible association between mutations of the β-catenin gene at putative GSK3β phosphorylation sites and nuclear accumulation has been reported in a variety of human malignancies, such as colorectal carcinomas with a normal APC gene, melanomas, and ovarian carcinomas (Rubinfeld et al, 1997; Palacios and Gamallo, 1998; Sparks et al, 1998).

*Received 6 March 2000*
*Revised 4 October 2000*
*Accepted 13 October 2000*

*Correspondence to:* Makoto Saegusa, Tel.: 042-778-8996;
Fax: +81-42-778-8441; E-mail, msaegusa @med.kitasato-u.ac.jp

The endometrial carcinoma is a common malignancy of the female reproductive tract. Although two distinctly different pathways of endometrial tumour development from hyperplasia (hyperplasia-carcinoma sequence) or postmenopausal endometrium, have been proposed (Deligdisch and Cohen, 1985), the underlying genetic mechanisms remain to be detailed. In this study, to clarify the possible role of β-catenin abnormalities, we investigated expression at the mRNA and protein levels, and mutations in exon 3 at or near the sites of targets of GSK3 β phosphorylation in normal, hyperplastic and malignant endometrium.

### MATERIALS AND METHODS

### Cases

A total of 199 cases of endometrial carcinoma (endometrioid type), surgically resected at the Kitasato University Hospital from 1988 to 1999, were consecutively investigated for immunohistochemistry, along with 141 normal samples (113 atrophic or proliferative and 28 secretory stages) distant from carcinomas. 37 cases of simple or complex hyperplasia without atypia and 32 of atypical hyperplasia were also examined. All tissues were routinely fixed in 10% formalin and processed for embedding in paraffin wax. Histological diagnoses were performed according to the criteria of the International Federation of Gynecology and Obstetrics (FIGO). The carcinoma cases comprised 128 grade (G) 1, 39 G2, and 32 G3 lesions. Areas of squamous differentiation (SqD) within tumours, including squamous metaplasia (SqM) and morules, were classified after Zaino et al (1991), the former being identified in 28 cases and the latter in 27. Of carcinoma cases available for investigation of clinicopathological factors, 143 were classified as

FIGO stage I and 56 as stages II–IV, while 118 demonstrated upper myometrial invasion (less than half of myometrial depth) and 81 lower invasion. For nodal status in pelvic cavity and para-aortic sites, 32 (16 of 139 stage I and 16 of 53 stages II–IV tumours) featured metastasis and 160 were without apparent metastasis.

Of 199 carcinoma cases, 73 tumours (43 G1, 18 G2, and 12 G3) as well as 44 normal endometrial samples were snap-frozen in liquid nitrogen and stored at −80˚C until use for mRNA expression, mutation, and Western blot analyses.

### Immunohistochemistry

Immunohistochemistry was performed using a combination of the microwave-oven heating and standard streptavidin-biotin-peroxidase complex (LSAB kit, Dako, Copenhagen, Denmark) methods. Briefly, slides were heated in 10 mM citrate buffer (pH 6.0) for three 5-min cycles using a microwave oven and then incubated overnight at 4˚C with anti-β-catenin monoclonal antibody (×1000 dilution, Transduction Lab, Lexington, NY). To confirm the specificity of binding, normal mouse serum (×500 dilution) was supplied instead of primary antibody as a negative control.

Immunostaining patterns were divided into two types, cell membrane and nuclear, with or without cytoplasmic, staining. For the membrane staining type, the immunointensity was subclassified into 4 groups in comparison with an internal control as follows: 0, negative; 1+, weak; 2+, moderate; and 3+, strong. The immunointensity for normal endometrium adjacent to carcinoma as the internal positive control was set as 3+. Positive immunoreactivity was concluded with either 2+ or 3+. Membrane labelling index (M-LI) values were calculated after examining all positive membrane signals from among at least 1000 cells in 5 randomly selected fields in sections containing typical areas within tumours for each case, using high-power (×40 objective and ×10 ocular) magnification. Nuclear labelling index (N-LI) values were also calculated in a similar manner, counting cells with nuclear staining. On the basis of N-LI values, the lesions were subdivided into three categories, in accordance with the criteria of previous studies (Kondo et al, 1999; Nhieu et al, 1999) with minor modifications, as follows: less than 1% N-LIs, more than 1% but less than 10%, and more than 10%. Cases with N-LI values more than 1% were considered as positive in line with the results for relation to gene mutations (Table 4).

### RT-PCR and Southern blot hybridization

Total cellular RNA was extracted using Isogen (Nippon Gene Co., Tokyo, Japan) and cDNA was synthesized from 5 μg of total RNA using RAV-2 reverse transcriptase (Takara, Shiga, Japan) in the presence of random primers (Takara) and a ribonuclease inhibitor (Takara) in a 20 μl reaction volume at 42˚C for 60 min. The tissue samples used included at least 70% tumour elements as confirmed by histopathological examination of adjacent sections to those taken for mRNA analysis.

For semi-quantitation of β-catenin gene expression, co-amplification with β-actin gene was carried out using 2 μl of cDNA solution and Taq polymerase (Takara) in a 20 μl volume. Specific primers for β-catenin (sense, 5′-CCAGCGTGGA-CAATGGCTAC-3′ at exon 2 and antisense, 5′-TGAGCTCGA-GTCATTGCATAC-3′ at exon 4) and β-actin (sense, 5′-TGCTATCCAGGCTGTGCTAT-3′ and antisense, 5′-GATG-GAGTTGAAGGTAGTTT-3′) were applied. The PCR procedure was performed with 30 cycles of denaturation at 94˚C for 0.5 min, annealing at 55˚C for 0.5 min and extension at 72˚C for 0.5 min, with a final extension time of 5 min. As a positive control, normal squamous epithelium of uterine crevix was used since it has been demonstrated to exhibit strong β-catenin expression (de Boer et al, 1999; Kimura et al, 1999). Water was supplied instead of template DNA for each examination as a negative control. The quality of the cDNAs was confirmed with GAPDH gene specific primers (forward, 5′-TGATGACATCAAGAAGGTGGTGAAG-3′; reverse, 5′- TCCTTGGAGGCCATGTAGGCCAT-3′).

A 2 μl aliquot of each PCR reaction mixture was electrophoresed in a 2.5% agarose gel and transferred to a Hybond N nylon membrane (Amersham, Tokyo Japan). Filters were hybridized overnight with digoxigenin-labelled specific probes for both β-catenin (5′-ATATTGATGGACAGTATGCAA-3′ at exon 4) and β-actin (5′-ACTGACTACCTCATGAAGATCCTCACCGAG-3′). Hybridization signals were detected with a DIG Luminescent Detection Kit (Boehringer Mannheim). The conditions used for hybridization, washing, and detection were in line with the manufacturer’s recommendations.

Analysis of hybridization signals was achieved with the computer software NIH Image version 1.58. The relative amounts were calculated by normalization to the hybridization signals for β-actin in each case: the value for mRNA signals for β-catenin was divided by that for the latter.

### DNA extraction and PCR

DNA was extracted and purified from 32 cases of atypical hyperplasia through proteinase K/phenol-chloroform treatment. Six serial 4 μm thick paraffin wax sections were cut from each case and reviewed microscopically, and areas with at least 80% atypical hyperplastic lesions were microdissected.

A 148 bp fragment of exon 3 of the β-catenin gene was amplified in hemi-nested PCR reactions. The first PCR conditions in a volume of 10 μl with common forward (5′-ATTTGATGGAGTTG-GACATGG-3′) and outer reverse (5′- TGTTCTTGAGTGAAG-GACTGA-3′) primers were as follows: 30 cycles of 94˚C for 0.5 min, 55˚C for 1 min, and 72˚C for 1 min. Two μl aliquots of reaction products were then used for a second PCR reaction in a volume of 20 μl with the same reaction conditions, using inner reverse (5′-TCTTCCTCAGGATTGCCTT-3′) primers. As a negative control, water was supplied instead of template DNA for each examination.

### Sequencing analysis

The remaining 18 μl aliquot of RT-PCR products for normal and carcinoma samples and 20 μl of the amplicons for atypical hyperplasias were purified from agarose gels using a QIAEXII gel extraction kit (Qiagen KK, Tokyo, Japan) and sequenced using ABI PRISM Dye Terminator Cycle Sequencing Ready Reaction Kit with AmpliTaq DNA Polymerase, FS (Perkin-Elmer, Foster City, CA), according to the manufacturers’ protocols. The DNA sequence was collected and analysed on an ABI Prism 310 automated DNA sequencer. The PCR primers were used for sequencing the two strands of the amplified products.

### Western blot assay

The remaining tissues of RNA extraction were homogenized in 0.01 M phosphate-buffered saline (PBS) solution and clarified by

*© 2001 Cancer Research Campaign*

centrifugation. 20 μg protein samples were separated by 10% sodium dodecyl sulfate (SDS)-polyacrylamide gel electrophoresis and then electroblotted (100 mÅ for one hour) onto Immobilon-P (Millipore, Tokyo, Japan). After blocking, the filters were incubated overnight at 4˚C with anti-β-catenin monoclonal antibody (×1000 dilution, Transduction Lab) and anti-actin monoclonal antibody (CP01, CALBIOCHEM, Cambridge, MA). As a positive control, normal uterine cervical squamous epithelium was also used. Reactivity was visualized using the Western Blot Chemiluminescence Reagent (NEN Life Science Products, Boston, MA).

### Statistics

Comparative data were analysed using the Mann-Whitney U-test, the Pearson's correlation coefficient and the chi-square test. The cut-off for statistical significance was defined as $P < 0.05$.

### RESULTS

### Immunohistochemistry

In normal endometrium, strong membrane β-catenin immunoreactivity was diffusely observed in glandular cells, showing similar average M-LI values between atrophic/proliferative (97.1 ± 4.1%, mean ±SD) and secretory (97.8 ± 3.1%) phases. The majority of stromal and myometrial components were negative, while endothelial cells in some capillaries showed moderate to strong membrane immunostaining (Figure 1 a,b,g,h). Nuclear staining was not found in any normal tissue elements (Table 1).

Membrane β-catenin immunoreactivity in hyperplastic lesions was similar to that in normal glandular components, while nuclear with negative or weak cytoplasmic immunostaining (N-LI ≧ 1%) was noted in 4 (10.8%) of 37 simple or complex and in 10 (31.3%) of 32 atypical hyperplasias (Figure 1 c,d,i,j), but the difference did not reach significant when subdivision was into three categories on the basis of the N-LI values (Table 1).

A variety of β-catenin staining patterns were observed in endometrial carcinomas, ranging from diffuse strong membrane staining to a complete lack (Figure 1 e,f,k,l). Nuclear, with or without cytoplasmic, immunostaining (N-LI ≧ 1%) was apparent in 55 (27.6%) of 199 carcinomas, being significantly related to the grade of histological malignancy (Table 1). In two cases with coexistence of carcinoma and atypical hyperplasia lesions, membrane immunoreactivity for β-catenin appeared to be decreased in the former as compared to the latter, while nuclear immunoreactivity was negative in both lesions.

Average LI values for β-catenin membrane staining were significantly decreased in the sequence leading from normal or non-atypical hyperplastic endometrium, through atypical hyperplasia,

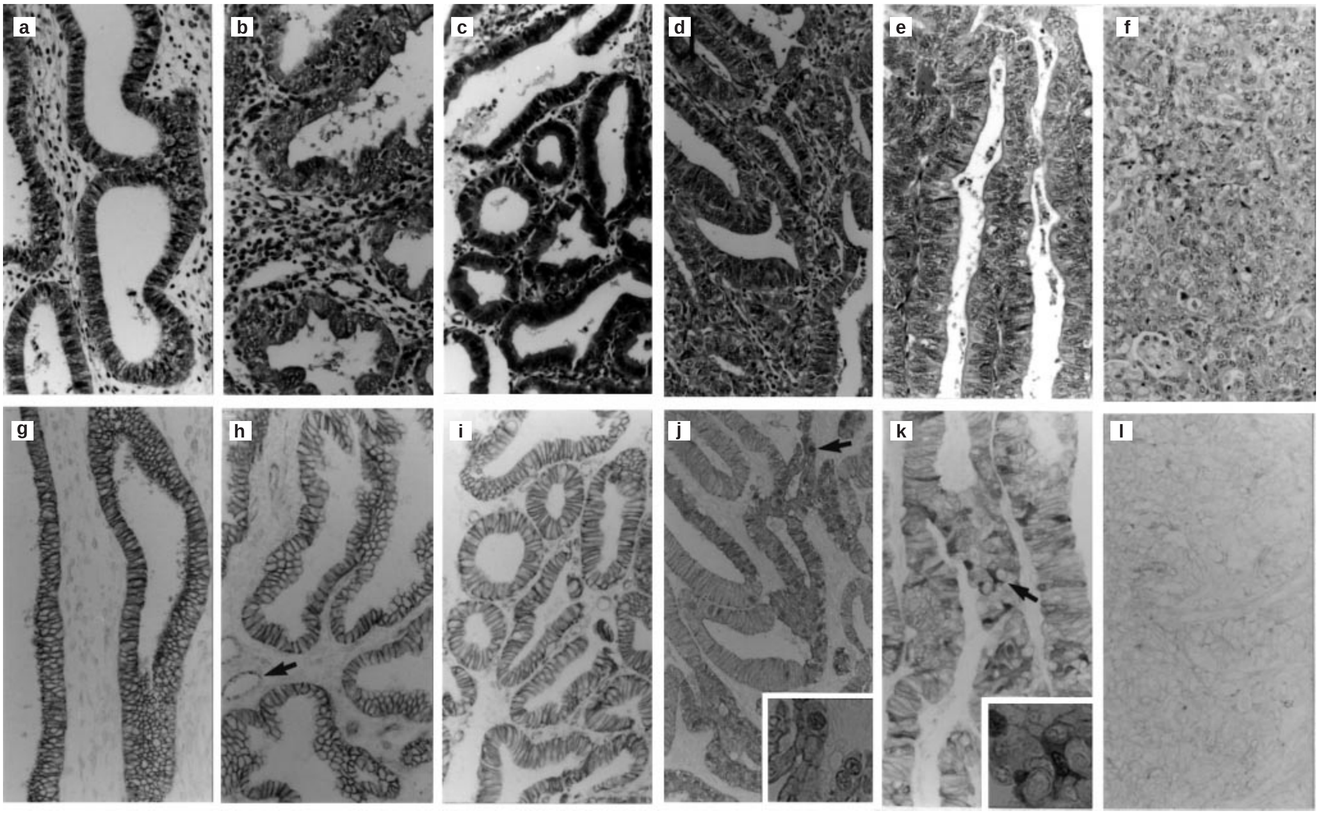

**Figure 1** Semiserial sections of normal, hyperplastic, and malignant endometrium. **a–f**) H&E staining. **g–l**) β-catenin immunohistochemistry. In proliferative (**a**) and secretory (**b**) phases of normal endometrium, strong β-catenin immunoreactivity is observed for cell membranes but not nuclei of glandular cells, while a lack of any immunoreaction is evident for stromal components (**g**, **h**), with the exception of endothelial cells in capillaries (**h**), indicated by the arrow. Strong membrane staining is also apparent in a complex hyperplasia (**c**, **i**), while the antibody against β-catenin also binds to nuclear sites in atypical hyperplasia (**d**, **j**). Note the strong nuclear and cytoplasmic but relatively weak membranous immunoreactivity in a grade 1 carcinoma (**e**, **k**) and weak membranous binding without nuclear accumulation in a grade 3 tumor (**f**,**l**). Higher magnification (×520) views of areas indicated by arrows are provided in the insets (**j**, **k**). Original magnification, ×200.

*© 2001 Cancer Research Campaign* 

**Table 1** Nuclear β-catenin expression in normal, hyperplastic, and malignant endometrium

| | β-Catenin nuclear staining (%) | | | | |
|---|---|---|---|---|---|
| | ***n*** | **N-LI<1%** | **1% ≦ N-LI<10%** | **10% ≦ N-LI** | ***P* value** |
| Carcinoma | | | | | |
| Grade 1 | 128 | 91 (71.1) | 25 (19.5) | 12 (9.4) | |
| Grade 2 | 39 | 27 (69.2) | 12 (30.8) | 0 | 0.039 |
| Grade 3 | 32 | 26 (91.3) | 2 (6.2) | 4 (12.5) | |
| Hyperplasia | | | | | |
| Non-atypical | 37 | 33 (89.2) | 4 (10.8) | 0 | 0.058 |
| Atypical | 32 | 22 (68.8) | 7 (21.9) | 3 (9.3) | |
| Mutation (+) | 3 | 0 | 3 (100) | 0 | 0.006 |
| (–) | 21 | 15 (71.4) | 3 (14.3) | 3 (14.3) | |
| Normal | 141 | 141 (100) | 0 | 0 | |

N-LI, nuclear labelling index; *n*, number of cases; Mutation, exon 3 mutation of the β-catenin gene in atypical hyperplasias. Statistical analysis was performed with the chi-square test.

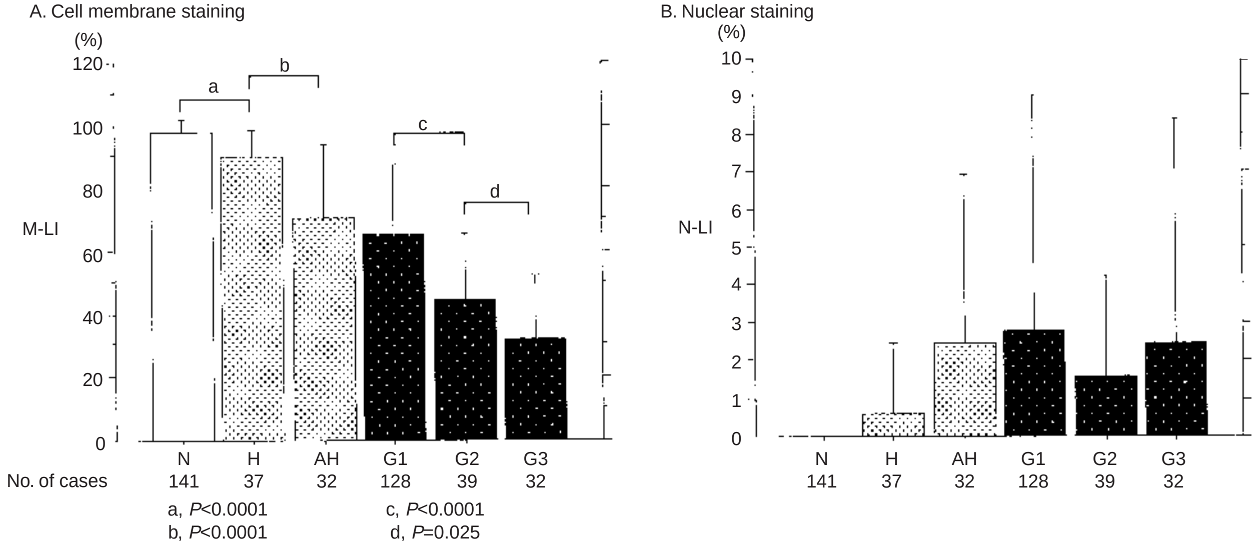

**Figure 2** Immunoreactivity score for cell membrane β-catenin staining. N, normal endometrium; H, non-atypical hyperplasia; AH, atypical hyperplasia; G, grade. The data are mean ± SD values. Statistical analysis was performed using the Mann-Whitney U-test.

to grade 3 carcinoma (Figure 2A), while there were no differences in N-LI values among non-atypical and atypical hyperplastic and carcinoma lesions (Figure 2B).

In both hyperplasias and carcinomas, the average M-LI values in nuclear immunopositive groups (N-LIs ≧ 1%) were significantly lower than in their negative counterparts (Figure 3).

The β-catenin antibody also bound to areas of SqD within tumours (Figure 4A), but average M-LI values, in particular in morules, were significantly lower than in carcinomatous areas, in contrast to significantly higher N-LIs (Figure 4B).

No apparent correlation between either membrane or nuclear immunopositivity and several clinicopathological parameters investigated was observed for the carcinoma category, with the exception of relation between M-LIs and degree of histological malignancy (Table 2).

### mRNA expression and analysis of β-catenin gene mutations

By co-amplification assay with β-actin gene, RT-PCR products of the β-catenin gene with the expected molecular weight (290 bp) were detected in all normal and tumour samples investigated, no small bands (less than 290 bp) being identified (Figure 5A). No differences in the relative mRNA intensity were observed. In addition, the mRNA expression levels were not related to any immunohistochemical parameters or clinicopathological factors investigated for endometrial carcinomas (data not shown).

Sequence analysis for the RT-PCR amplicons of β-catenin exon 3 revealed heterozygous substitution mutations in 16 (22.9%) of 70 informative tumour cases, whereas no mutations were noted in 12 normal samples. Data for endometrial cases with β-catenin mutations are summarized in Table 3. Mutations at potential phosphorylation target residues (serine/threonine at codons 33, 35, 37, 41 and 45) were detected in 14 (87.5%) of 16 cases, the remaining 2 cases being mutated at codon 34. The most common type of mutation was a C:G to T:A transition, present in 9 (56.3%) cases, with an A:T to C:G transition in 3, and a C:G to G:C transversion in 4 (Figure 5B). Mutations at codon 33 were also detected in 3 (12.5%) of 24 informative atypical hyperplasia cases, with a C:G to G:C transversion in 2 and a C:G to A:T transition in one case.

As shown in Table 4, β-catenin mutations were significantly correlated with grade 1 or 2 tumours, low M-LIs, high N-LIs, and

 *© 2001 Cancer Research Campaign*

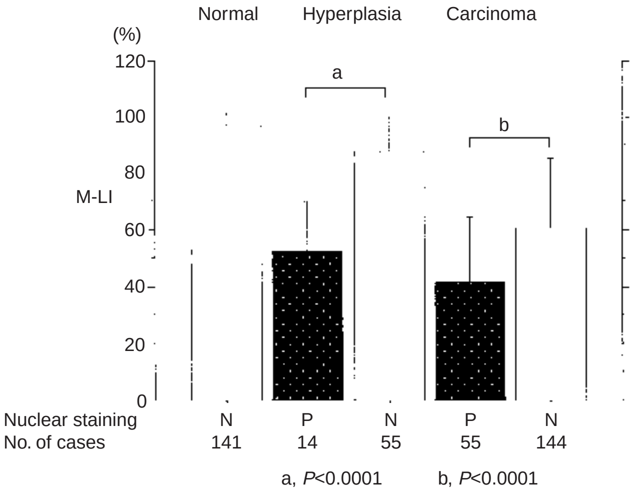

**Figure 3** Relation between β-catenin membrane and nuclear immunostaining. N, negative for nuclear immunopositivity; P, positive. The data are mean ± SD values. Statistical analysis was performed using the Mann-Whitney U-test negative lymph node metastasis. Positive relation to nuclear immunopositivity was also evident, as well as that of atypical hyperplastic lesions (Table 1), but there was no association with the relative mRNA intensity levels.

### Western blot assay

In cases available for Western blot analysis, bands of β-catenin showed a molecular weight of 92 kDa with or without several lower fragments, probably due to protein degradation, but truncated products were not observed (Figure 5C). Positive signals were detected in 29 (96.7%) of 30 normal samples and in 58 (85.3%) of 68 carcinomas (Table 3), no association with relative mRNA intensity, the presence of mutations, or immunohistochemical results being evident.

### DISCUSSION

In an endometrial cancer cell line, cell to cell aggregation and expression of E-cadherin, α- and β-catenin mRNAs have been reported to be significantly suppressed by estrogen with reversal by progesterone, suggesting regulation of the cadherin-associated cell adhesion system by sex steroid hormones (Fujimoto et al, 1996). Our results, however, did not show any differences in membrane β-catenin expression among atrophic, proliferative, and secretory phases in normal endometrium.

Nuclear β-catenin accumulation with or without mutations in exon 3 of the gene in atypical hyperplasia appeared to be higher than in simple or complex hyperplasias, the majority of the latter regressing or remaining stable, while approximately 30% of atypical hyperplasia progress to low-grade carcinomas (Kerman, 1985). Yoshinaga et al (1998) also proposed that atypical hyperplasias are at high risk for cancer development, whereas simple or complex hyperplasias are not, on the basis of microsatellite analyses. Considering the two proposed pathways of endometrial tumorigenesis (Deligdisch and Cohen, 1985), we therefore conclude that alteration in β-catenin expression may play an important role in a relatively early event in the endometrial hyperplasia-carcinoma sequence. Similar observations have been

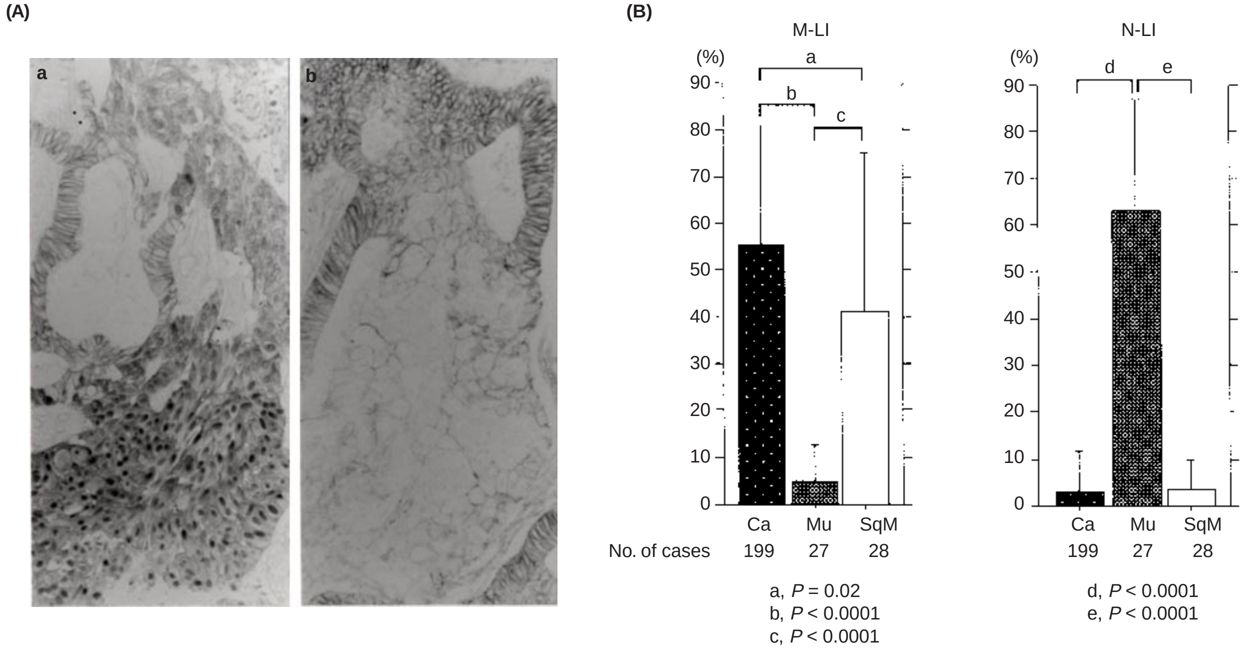

**Figure 4** (**A**) Immunoreactivity for β-catenin in areas of squamous differentiation within an endometrial carcinoma. (**A–a**) Note the strong nuclear immunopositivity with a weak membrane immunoreaction in a morule (lower half). (**A–b**) Relatively weak membrane staining in a squamous metaplastic area (lower half) as compared to the glandular portion. (**B**) Immunoreactivity scores for β-catenin membrane staining among carcinoma (Ca), morule (Mu), and squamous metaplastic (SqM) lesions. The data are mean ± SD values. Statistical analysis was performed using the Mann-Whitney U-test

**Table 2** Relation between either membrane or nuclear β-catenin immunoreactivity and clinicopathological factors in endometrial carcinomas

| | | Membrane LI | | Nuclear LI | |
|---|---|---|---|---|---|
| | *n* | m ± SD | *P* value | m ± SD | *P* value |
| Histology | | | | | |
| Grade 1 | 128 | 64.9 ± 22.4 | | 2.8 ± 6.2 | |
| Grade 2 | 39 | 44.6 ± 20.9 | | 1.6 ± 2.7 | |
| Grade 3 | 32 | 31.7 ± 20.8 | a | 5.1 ± 18.2 | NS |
| FIGO stage | | | | | |
| I | 143 | 57.6 ± 25.6 | | 2.8 ± 9.5 | |
| II/III/IV | 56 | 50.6 ± 24.5 | 0.08 | 3.3 ± 7.0 | 0.48 |
| Myometrial invasion | | | | | |
| Upper | 118 | 56.3 ± 26.1 | | 3.7 ± 11.0 | |
| Lower | 81 | 54.7 ± 24.5 | 0.62 | 1.8 ± 4.0 | 0.51 |
| LN metastasis | | | | | |
| Positive | 32 | 49.3 ± 25.0 | | 1.3 ± 3.1 | |
| Negative | 160 | 57.3 ± 24.9 | 0.09 | 3.0 ± 9.4 | 0.37 |

Ll, labelling index; m, mean; LN, lymph node; a, Grade 1 vs. Grade 2, $P < 0.0001$, Grade 1 vs. Grade 3, $P < 0.0001$, Grade 2 vs. Grade 3, $P = 0.025$; NS, not significant. Statistical analysis was performed with the Mann-Whitney U-test.

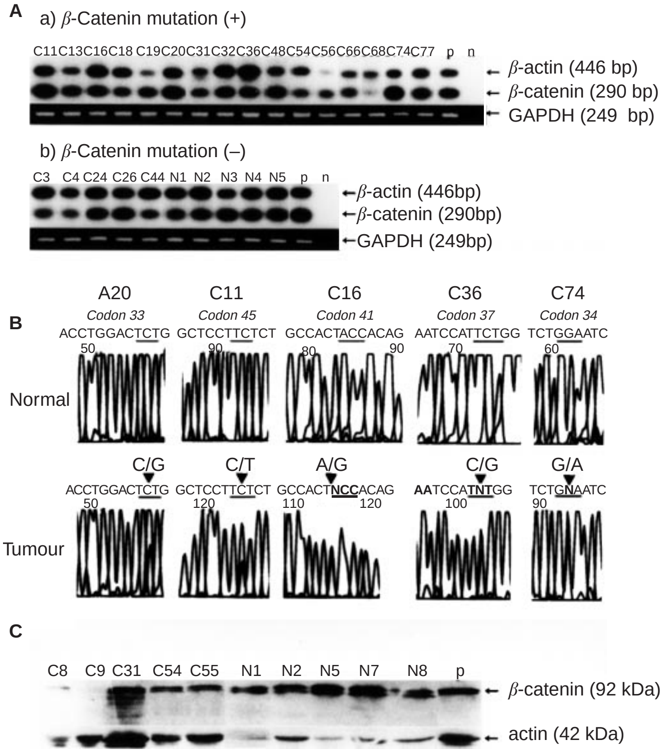

**Figure 5** (**A**) RT-PCR and Southern blot hybridization analysis for β-catenin mRNA in mutation positive (a) and negative (b) groups, with co-amplification of β-actin gene. Amplicons for GAPDH gene are also demonstrated as internal controls for each case. Case numbers for tumoirs with mutations correspond to those in Table 3. C, carcinoma cases; N, normal endometrial tissues; p, positive control (normal uterine cervical tissue); n, negative control (water). (**B**) Sequencing data for atypical hyperplasia (A20) and endometrial carcinomas (C11, C16, C36, and C74). (**C**) Western blot analysis of carcinoma (C) and normal (N) samples. After detection of β-catenin proteins, the filter was stripped to be incubated with an antibody against actin. p, positive control (normal uterine cervical tissue)

demonstrated for colorectal and ovarian endometrioid tumorigenesis (Palacios and Gamallo, 1998; Samowitz et al, 1999).

In this study, we identified mutations in exon 3 of the β-catenin gene in 16 (22.9%) of 70 endometrial carcinomas, in line with previous studies (Fukuchi et al, 1998; Kobayashi et al, 1999; Mirabelli-Primdahl et al, 1999). Although our mutation analysis was limited, it has been reported that mutations outside exon 3 of the β-catenin gene was infrequent, despite the entire coding sequence was analysed (Ilyas et al, 1997). In our series, the mutations in most cases (14 of 16) involved the loss of serines or threonines from GSK-3 β phosphorylation sites (codons 33, 35, 37, 41 and 45), as reported earlier (Fukuchi et al, 1998; Palacios and Gamallo, 1998; Garcia-Rostan et al, 1999; Mirabelli-Primdahl et al, 1999). Two cases showed a glycine to glutamic acid substitution at codon 34 which did not involve phosphorylation regions, but this abnormality is considered to interfere with β-catenin degradation by altering recognition sequences or tertiary protein structure (Sparks et al, 1998).

Although APC mutations may lead to stabilization or accumulation of β-catenin protein (Aberle et al, 1997; Orford et al, 1997), they are rare events in endometrial carcinomas (Jones et al, 1994; Berchuck and Boyd, 1995). In this study, β-catenin mutations in exon 3 was significantly related to low membrane and high nuclear immunoreactivity but not the relative mRNA intensity. Considering our findings for significant decrease of the membrane β-catenin expression together with the nuclear accumulation, gene mutations may be closely linked with an altered subcellular distribution rather than overexpression of mutated products in endometrial carcinomas. The finding of a possible reciprocal relation between loss of membrane expression and nuclear localization of β-catenin in colorectal carcinomas is interesting in this context (Hao et al, 1997). Our results that 6 carcinoma and 6 atypical hyperplasia cases without mutations also exhibited a distinct nuclear immunostaining may indicate the presence of other molecular pathways for β-catenin stabilization; for example, it has been reported that the wnt-1 oncogene promotes the accumulation of β- and γ-catenin (Hinck et al, 1994).

As another possible mechanism of β-catenin stabilization, somatic interstitial deletions of β-catenin exon 3, along with small transcripts

 © 2001 Cancer Research Campaign

**Table 3** Summary of data for endometrial carcinomas with β-catenin mutation

| Case no | Grade | mRNA level (/β–actin) | Mutated codon | Amino acid substitution | Western blot | Immunohistochemistry M-LI (%) | Immunohistochemistry N-LI (%) | Stage | Myometrial invasion | Lymph node metastasis |
|---|---|---|---|---|---|---|---|---|---|---|
| C11 | I | 1.42 | 45 (TCT to TTT) | Ser to Phe | P | 36.4 | 5.8 | I | Upper | Negative |
| C13 | I | 1.79 | 37 (TCT to TTT) | Ser to Phe | P | 53.7 | 4.4 | I | Lower | Negative |
| C16 | II | 0.92 | 41 (ACC to GCC) | Thr to Ala | P | 45.4 | 3.8 | I | Lower | Negative |
| C18 | I | 0.86 | 41 (ACC to ATC) | Thr to Ile | P | 50 | 9.8 | II | Upper | Negative |
| C19 | I | 2.82 | 37 (TCT to TGT) | Ser to Cys | P | 9.9 | 13.8 | II | Upper | Negative |
| C20 | I | 1.32 | 37 (TCT to TTT) | Ser to Phe | P | 27.8 | 4.8 | I | Upper | Negative |
| C31 | II | 0.91 | 33 (TCT to TGT) | Ser to Cys | P | 9.5 | 2.7 | II | Lower | Negative |
| C32 | II | 0.81 | 41 (ACC to GCC) | Thr to Ala | P | 30.2 | 5.3 | II | Upper | Negative |
| C36 | I | 0.65 | 37 (TCT to TGT) | Ser to Cys | P | 43.2 | 9.1 | II | Lower | Negative |
| C48 | I | 1.48 | 37 (TCT to TTT) | Ser to Phe | ND | 9.8 | 36.2 | I | Upper | NE |
| C54 | I | 0.81 | 34 (GGA to GAA) | Gly to Glu | P | 38.9 | 1.9 | I | Upper | Negative |
| C56 | I | 3.88 | 37 (TCT to GCT) | Ser to Ala | P | 52.1 | 6.2 | I | Upper | Negative |
| C66 | I | 1.49 | 45 (TCT to TTT) | Ser to Phe | P | 33 | 5.6 | III | Lower | NE |
| C68 | I | 0.42 | 37 (TCT to TGT) | Ser to Cys | P | 89.5 | 2.3 | III | Lower | Negative |
| C74 | I | 1.9 | 34 (GGA to GAA) | Gly to Glu | ND | 41.5 | 4.7 | I | Lower | Negative |
| C77 | I | 1.13 | 37 (TCT to TTT) | Ser to Phe | P | 40.2 | 3.2 | I | Lower | Negative |
| Normal EM | | 3.2 ± 6.0 (*n* = 44) | | | 29/30* | 97.5 ± 3.9 (*n* = 144) | 0 | | | |
| Carcinoma | | 1.7 ± 3.2 (*n* = 74) | | | 58/68* | 55.6 ± 25.4 (*n* = 199) | 2.9 ± 8.9 | | | |

no, number; EM, endometrium; P, positive; ND, not done; NE, not examined; *, total numbers investigated.

© 2001 Cancer Research Campaign

**Table 4** Relation between β-catenin mutation and clinicopathological factors and nuclear immunoreactivity in endometrial carcinomas

| | | β-catenin mutation | | |
|---|---|---|---|---|
| | **n** | **Positive (%)** | **Negative (%)** | ***P* value** |
| Histology | | | | |
| Grade 1 | 40 | 13 (32.5) | 27 (67.5) | |
| Grade 2 | 18 | 3 (16.7) | 15 (83.3) | |
| Grade 3 | 12 | 0 | 12 (100) | 0.048* |
| FIGO stage | | | | |
| I | 41 | 9 (22.0) | 32 (78.0) | |
| II/III/IV | 30 | 7 (23.3) | 23 (76.7) | 0.89* |
| Myometrial invasion | | | | |
| Upper | 29 | 8 (27.6) | 21 (72.4) | |
| Lower | 42 | 8 (19.0) | 34 (81.0) | 0.4* |
| LN metastasis | | | | |
| Positive | 15 | 0 | 15 (100) | |
| Negative | 50 | 13 (26.0) | 37 (74.0) | 0.027* |
| Membrane LI | | 38.2 ± 19.9 (*n* = 16) | 59.9 ± 24.3 (*n* = 50) | 0.002** |
| Nuclear staining | | | | |
| N-LI <1% | 49 | 0 | 49 (100) | |
| 1% ≦ N-LI<10% | 19 | 14 (73.7) | 5 (26.3) | |
| 10% ≦ N-LI | 3 | 2 (66.7) | 1 (33.3) | <0.0001* |
| Nuclear LI | | 7.5 ± 8.3 (*n* = 16) | 0.9 ± 2.8 (*n* = 50) | <0.0001** |
| Relative mRNA intensity | | 1.4 ± 0.9 (*n* = 16) | 1.8 ± 3.6 (*n* = 55) | 0.9** |

LN, lymph node; LI, labelling index; N-LI, nuclear labelling index; *, chi-square test; **, Mann-Whitney U-test.

and the truncated proteins lacking 76 amino acids, have been reported in colorectal carcinomas with wild type APC (Iwao et al, 1998). Such aberrant transcripts and proteins in our series, however, could not be demonstrated in any of tumours investigated, although we did not perform genomic DNA analyses.

With regard to the clinical implications of β-catenin abnormalities, colorectal adenomas with mutations have been demonstrated to be less likely to progress to larger adenomas and invasive carcinomas (Samowitz et al, 1999). In endometrial carcinomas, the alterations have been considered to be related to an early clinical stage with well differentiated histology or early onset of endometrial carcinomas (Fukuchi et al, 1998; Kobayashi et al, 1999). In addition to histological malignancy, we also found a significant correlation with no lymph node metastasis. This observation supports our conclusion of an association between β-catenin alterations and the endometrial hyperplasia-carcinoma sequence, since carcinomas developing with background of hyperplasia exhibit slow growth, spontaneous regression, and limited metastatic potential as compared to tumours arising from non-hyperplastic endometrium (Kurman et al, 1985). A lack of association between N-LI values and nodal metastasis may be due to the presence of tumours showing nuclear β-catenin accumulation without mutations of the gene.

We previously demonstrated aberrant DCC (deleted in colon cancer) and bcl-2 expression in morules as compared with SqM and primary tumour lesions (Saegusa and Okayasu, 1997; Saegusa et al, 1999). In this study, a high frequency of β-catenin nuclear localization was evident in morules but not SqM areas within tumours, indicating changes in expression of various molecules during development of morule foci.

A lack of correlations among mRNA, Western blot, and immunohistochemical findings was observed in this study. Possible reasons for this anomaly include the following: 1. contamination with endothelial cells expressing β-catenin; 2. degradation of protein products during sample preparation and processing due to protease activity; 3. differences in the protein fractions used in Western blot assay, since membrane β-catenin is mainly found in insoluble (membrane-bound) rather than soluble (cytoplasmic) fractions; 4. differences in sensitivity among the three assays. Discrepant results between levels of mRNA and protein have also been demonstrated for CD44 and DCC genes (Woodman et al, 1996; Horstmann et al, 1997). We therefore concluded that the immunohistochemical analysis is more accurate for assessment of actual levels of β-catenin protein, although PCR-based assays may be more sensitive.

In conclusion, the present study demonstrated a possible association between β-catenin alteration and endometrial tumorigenesis involving atypical hyperplastic lesions.

### REFERENCES

Aberle H, Bauer A, Stappert J, Kispert A and Kemler R (1997) β-catenin is a target for the ubiquitin-proteasome pathway. *EMBO J* **16**: 3797–3804

Beherens J, Kries JPV, Kuhl U, Bruhn L, Wedlich D, Grosschedl R and Birchmeier W (1996) Functional interaction of β-catenin with the transcription factor LEF-1. *Nature* **382**: 638–642

Berchuck A and Boyd J (1995) Molecular basis of endometrial cancer. *Cancer* **76**: 2034–2040

de Boer CJ, van Dorst E, van Krieken H, Jansen-van Rhiji CM, Warnaar SO, Fleuren GJ and Litvinov SV (1999) Changing roles of cadherins and catenins during progression of squamous intraepithelial lesions in the uterine cervix. *Am J Pathol* **155**: 505–515

*© 2001 Cancer Research Campaign*

Deligdisch L and Cohen CJ (1985) Histologic correlates and virulence implications of endometrial carcinoma associated with adenomatous hyperplasia. *Cancer* **56**: 1452–1455
Fujimoto J, Ichigo S, Hori M, Morishita S and Tamaya T (1996) Progestins and danazol effect on cell-to-cell adhesion, and E-cadherin and α- and β-catenin mRNA expressions. *J Steroid Biochem Mol Biol* **57**: 275–282
Fukuchi T, Sakamoto M, Tsuda H, Maruyama K, Nozawa S and Hirohashi S (1998) β-catenin mutation in carcinoma of the uterine endometrium. *Cancer Res* **58**: 3526–3528
Garcia-Rostan G, Tallini G, Herrero A, D′Aquila TG, Carcangiu ML and Rimm DL (1999) Frequent mutation and nuclear localization of β-catenin in anaplastic thyroid carcinoma. *Cancer Res* **59**: 1811–1815
Gumbiner BM (1996) Cell adhesion: the molecular basis of tissue architecture and morphogenesis. *Cell* **84**: 345–357
Hao XP, Tomlinson I, Ilyas M, Palazzo JP and Talbot IC (1997) Reciprocity between membranous and nuclear expression of β-catenin in colorectal tumours. *Virchows Archiv* **431**: 167–172
Hinck L, Nelson WJ and Papkoff JJ (1994) Wnt-1 modulates cell-cell adhesion in mammalian cells by stabilizing β-catenin binding to the cell adhesion protein cadherin. *J Cell Biol* **124**: 729–741
Horstmann MA, Posl M, Scholz RB, Anderegg B, Simon P, Baumgaertl K, Delling G and Kabisch H (1997) Frequent reduction or loss of DCC gene expression in human osteosarcoma. *Br J Cancer* **75**: 1309–1317
Ilyas M, Tomlison IPM, Rowan A, Pignatelli M, Bodmer WF (1997) β-catenin mutations in cell lines established from human colorectal cancers. *Proc Natl Acad Sci USA* **94**: 10330–10334
Iwao K, Nakamori S, Kameyama M, Imaoka S, Kinoshita M, Fukui T, Ishiguro S, Nakamura Y and Miyoshi Y (1998) Activation of the β-catenin gene by interstitial deletions involving exon 3 in primary colorectal carcinomas without adenomatous polyposis coli mutations. *Cancer Res* **58**: 1021–1026
Jones MH, Koi S, Fujimoto I, Hasumi K, Kato K and Nakamura Y (1994) Allelotype of uterine carcinoma by analysis of RFLP and microsatellite polymorphisms: frequent loss of heterozygosity on chromosome arms 3p, 9q, 10q, and 17p. *Cenes Chromosomes Cancer* **9**: 119–123
Kimura Y, Shiozaki H, Doki Y, Yamamoto M, Utsunomiya T, Kawanishi K, Fukuchi N, Inoue M, Tsujinaka T and Monden M (1999) Cytoplasmic β-catenin in esophageal cancers. *Int J Cancer* **84**: 174–178
Kobayashi K, Sagae S, Nishioka Y, Tokino T and Kudo R (1999) Mutations of the β-catenin gene in endometrial carcinomas. *Jpn J Cancer Res* **90**: 55–59
Kondo Y, Kanai Y, Sakamoto M, Genda T, Mizokami M, Ueda R, Hirohashi S (1999) β-catenin accumulation and mutation of exon 3 of the β-catenin gene in hepatocellular carcinoma. *Jpn J Cancer Res* **90**: 1301–1309
Kurman RJ, Kaminski PF and Norris HJ (1985) The behavior of endometrial hyperplasia: a long-term study of “untreated” hyperplasia in 170 patients. *Cancer* **56**: 403–412
Mirabelli-Primdahl L, Gryfe R, Kim H, Millar A, Luceri C, Dale D, Holowaty E, Bapat B, Gallinger S and Redston M (1999) β-catenin mutations are specific for colorectal carcinomas with microsatellite instability but occur in endometrial carcinomas irrespective of mutator pathway. *Cancer Res* **59**: 3346–3351
Molenaar M, Wetering MVD, Oosterwegel M, Peterson-Maduro J, Godsave S, Korinek V, Roose J, Destree O and Clevers H (1996) XTcf-3 transcription factor mediates β-catenin-induced axis formation in xenopus embryos. *Cell* **86**: 391–399
Morin PJ, Sparks A B, Korinek V, Barker N, Clevers H, Vogelstein B and Kinzler K W. (1997) Activation of β-catenin-Tcf signaling in colon cancer by mutations in β-catenin or APC. *Science* **275**: 1787–1790
Nhieu JTV, Renard CA, Wei Y, Cherqui D, Zafrani ES, Buendia MA (1999) Nuclear accumulation of mutated-catenin in hepatocellular carcinoma is associated with increased cell proliferation. *Am J Pathol* **155**: 703–710
Orford K, Crockett C, Jensen JP, Weissman AM and Byers SW (1997) Serine phosphorylation-regulated ubiquitination and degradation of β-catenin. *J Biol Chem* **272**: 24735–24738
Ozawa M, Baribault H and Kemler R (1989) The cytoplasmic domain of the cell adhesion molecule uvomorulin associates with three independent proteins structurally related in different species. *EMBO J* **8**: 1711–1718
Palacios J and Gamallo C (1998) Mutations in the β-catenin gene (CTNNB1) in endometrioid ovarian carcinomas. *Cancer Res* **58**: 1344–1347
Peifer M, McCrea PD, Green KJ, Wieschaus E and Gumbiner BM (1992) The vertebrate adhesive junction protein β-catenin and plakoglobin and the Drosophila segment polarity gene armadillo form a multigene family with similar properties. *J Cell Biol* **118**: 681–691
Rubinfeld B, Robbins P, El-Gamli M, Albert I, Porfiri E and Polakis P (1997) Stabilization of β-catenin by genetic defects in melanoma cell lines. *Science* **275**: 1790–1792
Saegusa M and Okayasu I (1997) Down-regulation of bcl-2 expression is closely related to squamous differentiation and progesterone therapy in endometrial carcinomas. *J Pathol* **182**: 429–436
Saegusa M, Hashimura M, Hara A and Okayasu I (1999) Loss of expression of the gene deleted in colon carcinoma (DCC) is closely related to histologic differentiation and lymph node metastasis in endometrial carcinoma. *Cancer* **85**: 453–464
Samowitz WS, Powers MD, Spirio LN, Nollet F, van Roy F and Slattery ML (1999) β-catenin mutations are more frequent in small colorectal adenomas than in larger adenomas and invasive carcinomas. *Cancer Res* **59**: 1442–1444
Sparks AB, Morin PJ, Vogelstein B and Kinzler KW (1998) Mutational analysis of the APC/β-catenin/Tcf pathway in colorectal cancer. *Cancer Res* **58**: 1130–1134
Woodman AC, Sugiyama M, Yoshida K, Sugino T, Borgya A, Goodison S, Matsumura Y and Tarin D (1996) Analysis of anomalous CD44 gene expression in human breast, bladder, and colon cancer and correlation of observed mRNA and protein isoforms. *Am J Pathol* **149**: 1519–1530
Yoshinaga K, Sasano H, Furukawa T, Yamakawa H, Yuki M, Sato S, Yajima A and Horii A (1998) The PTEN, BAX, and IGFIIR genes are mutated in endometrial atypical hyperplasia. *Jpn J Cancer Res* **89**: 985–990
Zaino RJ, Kurman R, Herbold D, Gliedman J, Bundy BN, Voet R and Advani H (1991) The significance of squamous differentiation in endometrial carcinoma. *Cancer* **68**: 2293–2302

© 2001 Cancer Research Campaign
